# Balancing resources and responsibility: managers’ perspectives on promoting infection prevention behaviours in hospital settings

**DOI:** 10.1108/LHS-02-2025-0022

**Published:** 2025-11-11

**Authors:** Lisa Arvidsson, Maria Lindberg, Bernice Skytt

**Affiliations:** Department of Caring Sciences, Faculty of Health and Occupational Studies, University of Gävle, Gävle, Sweden, and Centre for Research and Development, Uppsala University/County Council of Gävleborg, Gävle, Sweden; Department of Caring Sciences, Faculty of Health and Occupational Studies, University of Gävle, Gävle, Sweden, and Department of Public Health and Caring Sciences, Uppsala University, Uppsala, Sweden

**Keywords:** Compliance, Hospital managers, Infection prevention behaviours, Managerial strategies, Nursing staff, Qualitative research, Reflexive thematic analysis, Working conditions

## Abstract

**Purpose:**

The purpose of this study is to explore hospital managers’ experiences and reflections concerning the influence of working conditions on nursing staff’s infection prevention behaviours and strategies used by the managers to promote infection prevention work among staff.

**Design/methodology/approach:**

The qualitative study uses a reflexive thematic analysis. Six first-line and five second-line managers at surgical and orthopaedic hospital units were interviewed.

**Findings:**

Reflecting a balance between resources and responsibility in promoting infection prevention behaviours, four themes were generated: (1) being attentive to staff needs and taking action in a changing healthcare environment, where managers adjust working conditions to minimise interruptions and manage workload; (2) bultivating a positive work climate for both the team and the individual, emphasising team collaboration and role modelling in infection prevention; (3) providing resources for knowledge development and understanding, including appointing hygiene representatives and promoting accessible infection control information; and (4) promoting personal responsibility for compliance and infection-safe workflows, highlighting staff responsibility regardless of working conditions.

**Originality/value:**

This study provides new insights into hospital managers’ perspectives on how working conditions influence nursing staff’s infection prevention behaviours and the strategies managers use to support compliance. Unlike prior research focused on frontline staff, this reflexive thematic analysis highlights the managers’ role in balancing organisational support with staff accountability, offering valuable insights into infection control in complex healthcare environments.

## Introduction

Healthcare managers hold responsibility for and possess the mandate to influence the working conditions of staff (Cummings *et al.*, 2018). Working conditions refer to the circumstances under which work is carried out and encompass a wide range of factors, from physical conditions to psychosocial elements and working hours. These are aspects of the workplace that can be altered and improved (International Labour Organization, 2024). The healthcare sector and its workforce must continuously adapt to societal, demographic, technological and pharmacological changes (International Labour Organization, 2017). Recent research shows that working conditions in healthcare settings are often inadequate, contributing to high turnover rates. Factors such as insufficient organisational support, inadequate staffing levels and high workloads lead to dissatisfaction among staff and are reasons why many choose to leave their positions (Leary *et al.*, 2024). For those working in inpatient care, where 24/7 services are provided, additional burdens arise from shift work, including night shifts and overtime is often used to cover staff shortages, further increasing the strain on existing staff (International Labour Organization, 2017).

In Swedish healthcare, first-line managers (FLMs) and second-line managers (SLMs) share responsibility for staff working conditions and patient safety. The FLM is the manager closest to the nursing staff. Nursing staff, in this study, refers to registered nurses and assistant nurses, who are the primary caregivers involved in direct patient care. FLMs are responsible for overseeing day-to-day operations, administrative tasks, staff coordination and decision-making focused on the needs of the unit. SLMs have overarching responsibility for the healthcare service, ensuring compliance with regulations related to both staff welfare and patient safety. SLMs are also responsible for providing the necessary resources and organisational structures to fulfil these obligations. Furthermore, systematic measures must be taken to prevent patient harm (Swedish Patient Safety Act SFS 2010:659, 2010). Managers themselves also face challenging working conditions, including role ambiguity, lack of support, high stress, limited resources and financial pressures (Berghout *et al.*, 2017; Labrague *et al.*, 2018).

Healthcare-associated infections (HCAIs) are infections acquired by patients due to the care processes, which patients did not have upon admission (World Health Organization, 2011). They represent the most common adverse event worldwide and pose a serious threat to patient safety (Haque *et al.*, 2018; World Health Organization, 2011). These infections contribute to increased morbidity and mortality, extended hospital stays and substantial societal costs, highlighting the importance of preventive efforts (Haque *et al.*, 2018). While factors such as length of hospitalisation, patient immune status, age and invasive procedure influence the risk of HCAIs (World Health Organization, 2011), infection prevention behaviours among healthcare staff are also crucial (Loveday *et al.*, 2014). According to a systematic review and meta-analysis, an estimated 35%–55% of HCAIs could be prevented through multifaceted interventions explicitly aiming to enhance infection prevention behaviours among staff (Schreiber *et al.*, 2018). Managers have a central role in the workplace and research shows that visible and engaged managers help staff overcome barriers such as resource constraints and high workloads through effective communication, while also fostering a safety culture and promoting compliance with infection prevention practices (McAlearney *et al.*, 2022).

Preventing HCAIs is a global patient safety priority (World Health Organization, 2021). A recent WHO-led Delphi study highlighted leadership as one of the highest-priority areas for future research, underlining the need to examine how managers influence hygiene practices (Tartari *et al.*, 2025). This study addresses that gap by exploring how hospital managers understand and promote infection prevention while navigating their dual responsibility for both staff working conditions and patient safety.

### Aim

The aim of this study was to explore hospital managers’ experiences and reflections concerning the influence of working conditions on nursing staff’s infection prevention behaviours and strategies used by the managers to promote infection prevention work among staff.

## Methodology

### Study design

We conducted a qualitative study using reflexive thematic analysis (Braun and Clarke, 2022). The reflexive thematic analysis reporting guidelines (RTARG) guided the reporting and overall presentation of the manuscript (Braun and Clarke, 2024).

### Context and participants

The setting was selected based on a previous study conducted by the research team (Arvidsson *et al.*, 2021). FLMs and SLMs from four surgical and four orthopaedic hospital units across Sweden, representing community hospitals, district hospitals and university hospitals in different regions, were invited to participate in the study. No inclusion criteria were formulated. Instead, individuals who held managerial positions at the units at the time of data collection were invited to participate, eight FLMs (the managers of each respective unit) and six SLMs, two SLMs oversaw two FLMs each, Eleven managers (6 FLMs and 5 SLMs) agreed to participate, with the majority being female (4 FLMs, 4 SLMs). All FLMs were registered nurses, while SLMs included three registered nurses and two physicians. FLMs supervised an average of 53 employees (range: 35–75), while SLMs oversaw an average of 251 employees (range: 74–350), reflecting their broader operational responsibilities. Four FLMs worked in shared leadership arrangements, while each SLM held an independent leadership role. All the managers had either completed a leadership education or were currently enrolled. Tenure at the current unit ranged from 0.1 to 6 years (mean: 1.6 years for FLMs, 2.1 years for SLMs). All had prior clinical experience and most had previous managerial experience (5 FLMs, 4 SLMs). Notably, three SLMs had previously worked as FLMs.

Regarding the hospital units represented by the managers, the units had between 16 and 24 patient beds available. All but one unit had access to additional beds that could not be opened due to staff shortages. Room structures varied: one department had only single-patient rooms, one had a mix of single, double and four-bed rooms and the others had single and double rooms. In daily operations, the nursing staff worked either in pairs consisting of a registered nurse and an assistant nurse or in teams comprising one registered nurse and two assistant nurses.

### Data generation

Data were collected between September 2023 and February 2024 through individual interviews, conducted by the first author, either via video link or in person, lasting 32–57 min. A semi-structured interview guide with open-ended questions and probes was used. Questions were developed based on findings from three previous studies by the research team, exploring relationships between infection prevention behaviours and working conditions (Arvidsson *et al.*, 2021, 2023, 2025). The interviews began with background questions, followed by a broad question asking the managers to describe how they believe working conditions influence nursing staff’s infection prevention behaviours. Managers were also asked to describe strategies for promoting infection prevention through working conditions. All interviews were audio recorded, with only the manager and interviewer (LA) present.A pilot interview was conducted with an SLM (former FLM), after which the broad question was moved to the beginning, as it had previously been placed at the end. This interview was excluded, as the intention was to include only managers from the previously involved units.

### Positioning

Given the aim of the study, a contextualist perspective was adopted. In our research group, there is extensive knowledge and practical experience in both qualitative research and infection prevention work.

### Data analysis

Drawing on my expertise in the field, I (LA) used Braun and Clarke’s most recent articulation of reflexive thematic analysis (2022, 2024). This approach acknowledges the active role of the researcher and recognises that the researcher’s background shapes interpretation. While I conducted the analysis, the research team engaged in critical discussions throughout the process. These dialogues provided reflexive engagement that enhanced interpretive depth and transparency, consistent with the principles of reflexive thematic analysis. The six phases of reflexive thematic analysis guided the process: familiarisation, coding, generating themes, reviewing, refining and writing. The process was iterative rather than linear. Shortly after each interview, I listened to the recordings and noted key points. The recordings were transcribed using Amberscript, an online automatic transcription tool (Amberscript Transcription services, 2025). The transcripts were checked for accuracy and identifying information was removed. Since I conducted all the interviews, I was already familiar with the data. However, to deepen this familiarisation, the transcribed interviews were read multiple times. Relevant data were systematically coded, with codes noted alongside transcripts and revised as the analysis progressed.

The subsequent phase involved searching for larger patterns across the data, with the codes being organised and collated into topic areas. The topic areas were compiled in a separate document, grouping those with commonalities together and creating a “map” using a PowerPoint file for flexibility. Importantly, even though previous research shaped the development of the interview guide, it did not dictate the analysis. The analysis was explorative, grounded in the data and not driven by pre-existing theories or frameworks.

From the collated topic areas, initial themes were formulated. First, I grouped all data related to the same topic area together. However, as the process progressed, we became increasingly convinced of the heterogeneity across data. To capture these divergences, data were reorganised. For instance, some managers emphasised the importance of psychosocial working conditions in shaping nursing staff’s infection prevention behaviours, noting how staff adopt the behaviours of others. In contrast, other managers argued that staff hold personal responsibility for their behaviours, independent of their colleagues’ actions. These differing perspectives were thus presented under separate themes, to avoid the creation of “topic summaries” (Braun and Clarke, 2022). In the analysis, FLM and SLM perspectives were treated together, as the focus was on overarching managerial views rather than subgroup comparisons.

To ensure that the analysis remained grounded in the data, I continually returned to the original transcripts and as the analysis progressed, the interpretations became increasingly abstracted. All authors participated in the final naming of themes and discussed whether an overarching theme could be generated. However, given the diversity of data, it was decided not to force the development of an overarching theme. According to Braun and Clarke (2022), reflexive thematic analysis does not necessitate or encourage the creation of an overarching theme, as attempting to force one can result in oversimplification or a loss of the complexity of the data. Instead, the study title was chosen to reflect the results as a whole.

### Ethics

This study was conducted in accordance with applicable ethical rules and national laws. The *Swedish* Ethical Review Authority approved the study protocol (reg. no. 2023-00408-01). The research adhered to the ethical principles outlined in the Declaration of Helsinki (World Medical Association, 2013). Participation was voluntary, with the option to withdraw at any time. Prior to the interviews, the managers received both written and verbal information. Confidentiality was ensured throughout the process and informed consent was obtained from all participating managers before the interviews.

## Reporting of the data analysis

**Figure 1.**
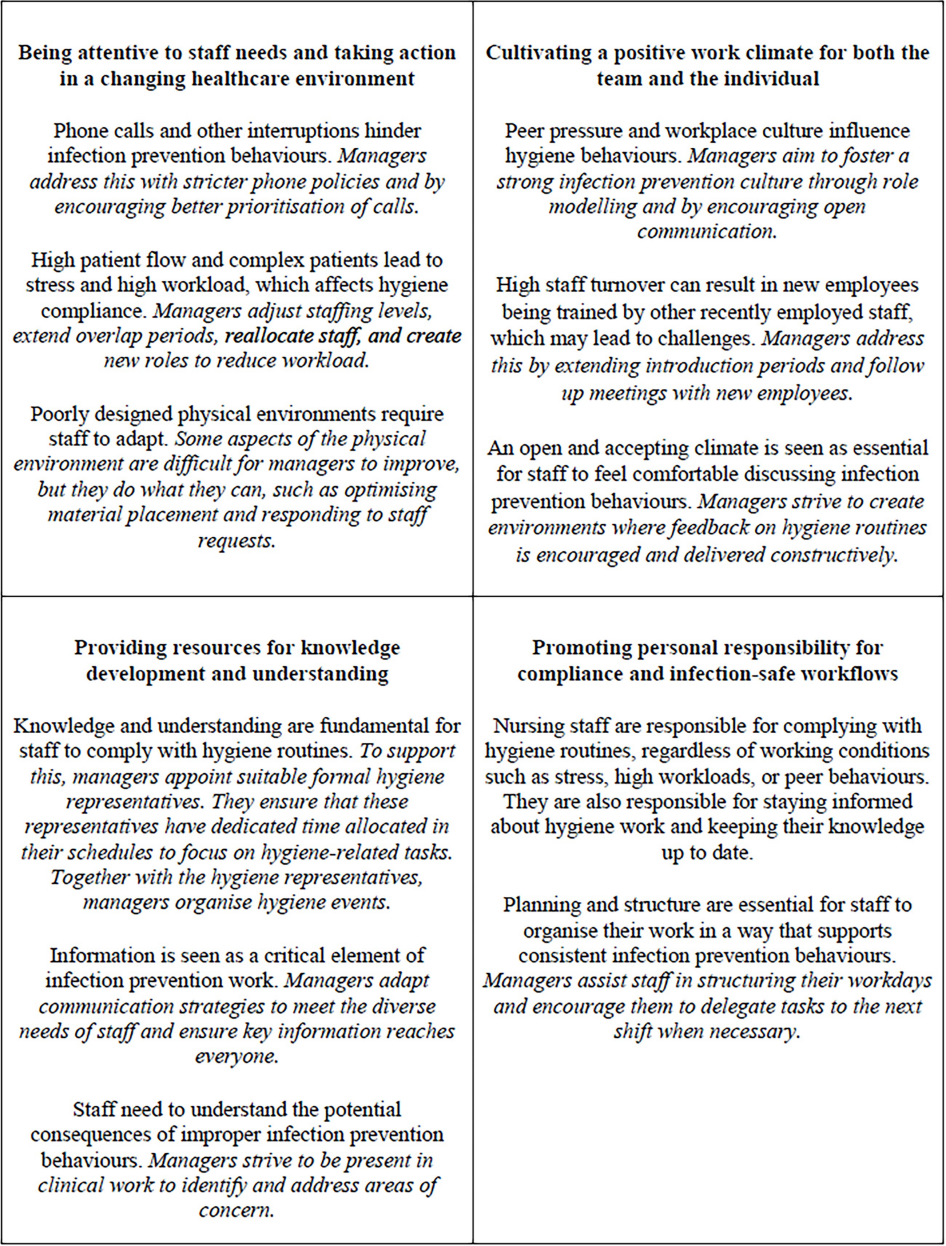
The figure illustrates the four themes (text in bold) generated from the study. Key aspects of both research question 1–the managers’ experiences regarding how working conditions influence nursing staff’s infection prevention behaviours (normal text) and research question 2 – strategies to promote infection prevention work among staff (text in italics) are presented under the name of each theme **Source:** Authors’ own work

Four themes were generated. To illustrate these and enhance readability, the theme names and key aspects from both research questions 1 and 2 are presented in Figure 1. The results reveal a dynamic interaction between organisational structures and the psychosocial culture and highlight the challenges that arise from the unpredictable context in which nursing staff operate. The managers identified several strategies they use to create working conditions that support infection prevention behaviours among nursing staff, while they also emphasised the personal responsibility of nursing staff in maintaining infection prevention behaviours, regardless of working conditions – highlighting the ongoing balance between responsibility and resources. To bring the findings to life and following the recommendations in RTARG (Braun and Clarke, 2024), the results from the analysis are presented in relation to other relevant research and theories. No distinction has been made between FLMs and SLMs in the narrative, but the specific role of the manager is indicated in each citation.

### Being attentive to staff needs and taking action in a changing healthcare environment

The managers described the hospital as a challenging workplace where unpredictable events frequently disrupt routines and require staff to adapt. According to the managers, such situations include events such as emergencies and interruptions. Interruptions were described as widespread in nursing work and the managers highlighted work phones as a frequent source of interruptions, particularly for registered nurses, which they believe negatively impacts working conditions for nursing staff and thus also risks having a negative effect on infection prevention behaviours:

Because the phones ring a lot […] it’s not just relatives who want an update. It is also coordinators, surgery schedulers, I call, the secretary calls, the lab calls. What I mean is that it’s really easy to just stick your gloved hand in your pocket to fish out a phone (Manager 5, FLM).

This extends findings from our previous work, which identified how interruptions negatively impact infection prevention behaviours (Arvidsson *et al.*, 2021), by providing managerial reflections on underlying causes and practical solutions. The managers described several strategies they use to improve working conditions for the nursing staff and reduce interruptions. For instance, reminding staff that it may be preferable to take a message and return calls at a more suitable time and implementing stricter phone policies.

Workload was described as another critical concern. The managers described how shorter hospital stays, higher patient flow and more complex conditions among patients have increased the workload for nursing staff. They associated high workloads with stress among staff and stated that stress is a working condition that affects infection prevention behaviours:

Stress is a big part of it. If there is a high workload and a lot of stress, you might skip certain hygiene steps because you think it will be quicker (Manager 1, FLM).

Given the high patient flow and fast work pace, the managers acknowledged their responsibility to ensure a reasonable workload for their staff by adjusting staffing levels, both in the long and short term. Sometimes, they need to call in additional staff when the workload is exceptionally high, though this is not always easy. They also described how they sometimes reallocate existing staff resources. Several managers stated that they have adjusted baseline staffing levels to meet the changing demands:

We have noted that in the evenings, the pace and pressure are at least as high as during the day, so we should have the same staffing then. You might think things would be a little calmer in the evenings, but that is not the case. Then we have to have the same number of assistant nurses on duty then. You have to keep track of staffing level so that it works (Manager 2, SLM).

Further, some managers aimed to reduce workload by extending overlap periods between shifts, reallocating tasks and introducing support roles such as receptionists or coordinators. These actions mirror findings from a recent review linking work-related stress to patient safety risks, which emphasises that organisational strategies such as increasing staffing levels and redistributing responsibilities are more effective in addressing the root causes of stress than individual interventions like mindfulness (Li *et al.*, 2024).

This finding about stress and high workload can be understood through the lens of the health belief model (Glanz *et al.*, 2015), which highlights how perceived barriers, such as stress and time pressure, can hinder health-related behaviours. These barriers may prevent individuals from complying with routines, even when they recognise the benefits of them. Stress is well known to be widespread in healthcare and high workload and time constraints are well-documented implementation barriers in infection prevention (Houghton *et al.*, 2020). In this context, managers play a crucial role in helping staff overcome such barriers, something healthcare staff themselves emphasise as important (Houghton *et al.*, 2020). Their role is central not only in daily practice but also in shaping organisational conditions through staffing, resource allocation and policy decisions. From a systems perspective, this reflects what the Systems Engineering Initiative for Patient Safety (SEIPS) 2.0 model emphasises: healthcare work is shaped by interactions between multiple system elements, such as the organisation, tasks, tools and environment, which together influence staff behaviours and patient outcomes (Holden *et al.*, 2013). The managers’ quotes in this theme illustrate how these roles operate at different levels: FLMs, with their close proximity to staff, recognise the everyday pressures and interruptions that challenge infection prevention, while SLMs act higher in the organisational hierarchy, responsible for adjusting staffing policies. Both levels of leadership are therefore needed to align working conditions with infection prevention goals.

The managers also highlighted the physical care environment as crucial to infection prevention. While they noted that the design of facilities is difficult for them to influence, they emphasised their responsibility to ensure that materials such as hand disinfectants and aprons are easily accessible.

### Cultivating a positive work climate for both the team and the individual

This theme captures how managers perceive that collegial interactions and the workplace climate and culture influence nursing staff’s infection prevention behaviours. They highlight that peer pressure plays a crucial role, as it is easy for staff to adopt the behaviours of others, especially senior colleagues or influential team members and this can happen both consciously and unconsciously. From behavioural theory, it is well established that people adjust their actions to perceived peer norms, particularly under uncertainty and that role models and authority figures strongly shape these norms (Cialdini, 2009). International research also shows that the impact of safety culture is context dependent. For example, a newly published study in Jordanian hospitals found that nursing staff working in organisations with clear infection control policies and a supportive culture reported higher compliance with hygiene practices (Harb *et al.*, 2025). In our study, managers stressed the importance of fostering a strong infection prevention climate, not only by shaping organisational conditions but also by serving as role models themselves. This resonates with Cialdini’s (2009) view that authority figures strongly shape group norms:

If you are not a good role model as a manager regarding hygiene, then it is difficult to demand the same from your staff (Manager 4, SLM).

This quote demonstrates that managers recognise the influence of their own actions on staff behaviour and underscoring leadership as a central driver in cultivating a supportive workplace culture. The managers further emphasised the importance of quickly educating new employees on correct infection prevention practices and integrating practical training in this process. Some have extended the introduction period for new employees after identifying this as a need. Additionally, the managers describe how they conduct follow-up meetings with nursing staff during their introduction and acknowledge that this process sometimes needs to be tailored to the individual. As leaders, the managers agree that it is their responsibility to ensure that new employees receive the introduction they need. At the same time, they reflect on the difficulties associated with the high staff turnover that many managers experience within their units:

There is kind of a knowledge gap among supervisory staff […]. It may be that we have lost the culture bearers in the workplace. Those who kind of hold up the walls and show what the routines are. It kind of spreads by word of mouth (Manager 11, FLM).

This quote not only highlights the concern about a knowledge gap among staff responsible for training but also suggests that this can lead to a negative shift in workplace culture. High turnover and staff shortages risk undermining the continuity needed to sustain safe care. The WHO reports a global shortage of registered nurses (World Health Organization, 2020). However, WHO, Organisation for Economic Co-operation and Development (OECD) and leading researchers stress that training more nurses will not solve the problem. The core challenge is retention: too few are willing to remain under current working conditions. Addressing this requires substantial improvements in the work environment rather than focusing solely on increasing supply (OECD, 2023; World Health Organization, 2020). In this light, managers’ efforts to provide thorough introductions and support experienced staff as “culture bearers” become crucial strategies for safeguarding infection prevention practices. As part of their strategy, the managers sought to foster an open and supportive environment, encouraging staff to voice their perspectives and framing feedback as constructive. Yet it remains uncertain whether such measures are sufficient, highlighting the need to prioritise healthcare working conditions at higher system and political levels.

### Providing resources for knowledge development and understanding

It doesn’t matter how much I tell someone to do something if they don’t have an understanding of why they need to do it a certain way (Manager 6, FLM).

This statement captures the essence of the theme: understanding is a cornerstone of infection prevention behaviours and requires not only knowledge of routines but also an understanding of their rationale. As framed by the health belief model (Glanz *et al.*, 2015), behaviour is shaped by perceived risk severity and benefits of action, highlighting the importance of understanding potential consequences of non-compliance. To ensure this understanding, the managers described several strategies. A key organisational measure was the appointment of formal hygiene representatives within their units. These representatives disseminate knowledge, update routines and perform local hygiene assessments which can assist both the managers and the hygiene representatives in identifying areas of non-compliance and determining where interventions may be needed. The managers stressed the importance of selecting suitable individuals for these roles and providing them with the necessary resources, including allocating time in their work schedules to focus on hygiene-related tasks and supporting them in organising activities such as hygiene weeks with training, lectures and competitions, for example, quizzes.

One of the aspects that the managers identified as central to understanding infection prevention work is the availability of accurate and accessible information and routines. The managers describe it as their responsibility to ensure that information reaches the staff but note that this can sometimes be challenging, as one manager described:

But everyone is so different. So if you as a manager think, ‘Yes, I’ve sent out emails, I put it on the bulletin board’. You’ve done that and yet there are some people who have never heard it [the information]. So the challenge is to reach all staff in a way that you can best reach them. Some want it to be as simple as possible and some want more detail. As a manager, you have a challenge in getting the information to everyone in the right way (Manager 9, SLM).

The managers noted that when compliance with hygiene routines declined among staff, it was often linked to gaps in knowledge and many observed that the level of knowledge among staff varies significantly. They described that nursing staff undergo Web-based hygiene training at the start of employment and at many units also annually thereafter. However, research shows that such approaches may be of limited value if staff engage passively and the effectiveness depends on context and design (Regmi and Jones, 2020). In contrast, simulation-based training, a more interactive approach, has shown promising results. A recent systematic review concluded that such training can effectively develop and improve non-technical skills, including communication, situational awareness and decision-making within interprofessional healthcare teams, with potential for broader application across healthcare systems (Pucer *et al.*, 2025).

To increase knowledge, the managers emphasised the importance of discussing the potential consequences of not complying with hygiene routines. They also recognised that it is their responsibility to ensure that nursing staff have the necessary competence, which can be facilitated by ensuring that they are present in the workplace:

And the fact that I am present and involved in care and see the shortcomings so that we can actually work on that too. I am also responsible for the staff. If someone says that they need competence, it is important that I meet that need (Manager 7, FLM).

Finally, managers described that when knowledge gaps among themselves or the hygiene representatives occurred, they sought support from the hospital’s infection control unit. In an older yet still highly relevant article by leading scholars in infection prevention, Gould and colleagues et al. (2016) argue that leadership is not only about formal roles but also about creating the conditions for others to take responsibility, something the managers in our study actively sought to do. They further highlight that managers must balance hierarchy with flexibility and local adaptation (Gould *et al.*, 2016). To conclude, while the ultimate responsibility for both patient safety and creating healthy working conditions rests with managers, they should also encourage and enable staff to take initiative and show leadership in infection prevention work.

### Promoting personal responsibility for compliance and infection-safe workflows

This theme differs from the previous three. Here, managers stated that nursing staff are responsible for complying with hygiene routines regardless of working conditions and factors such as stress or a heavy workload cannot be used as an excuse for neglecting this responsibility:

Of course, you could argue that it is the workload that makes you need to take shortcuts. I don’t think that’s the case. That’s not where the time savings are if you, for example, sanitise your hands or not (Manager 3, SLM).

Managers argued that staff working in acute care units must be able to manage stress without compromising compliance and that it is each individual’s duty to stay updated and maintain knowledge about hygiene routines. Furthermore, even if colleagues are careless, staff must still act responsibly:

It is important to take responsibility yourself. It is part of your duties to comply with hygiene routines, simple as that (Manager 7, FLM).

Regardless of working conditions, managers state that staff need to organise and plan their tasks to maintain infection prevention behaviours, thereby taking responsibility for and shaping their own and their colleagues’ working conditions. This applies both to specific tasks and to the workday as a whole and includes, for example, preparing materials before entering patient rooms to minimise movements in and out, planning the workday as a team and clarifying responsibilities during coordination meetings to reduce interruptions. They noted that poor planning could explain lapses in infection prevention. Managers described how they supported staff by assisting in task organisation, encouraging handovers to the next shift when necessary and underscoring the importance of completing tasks to safeguard infection prevention:

It is about me, as a registered nurse or assistant nurse, completing my task. […]. To be honest, we need to work on ensuring that individuals take the lead in their own work and take responsibility for completing their tasks (Manager 8, SLM).

Compared to earlier themes, which largely emphasised organisational strategies to support staff, this theme stands out in placing responsibility for infection prevention primarily on the individual. While managers stressed that every staff member has a duty to comply with routines regardless of workload, this framing risks overlooking the systemic and organisational factors that shape behaviour. From a systems perspective, this individualised view of patient safety work is increasingly regarded as outdated. Since the landmark report *To Err is Human* (Institute of Medicine, 2000), there has been a clear shift in patient safety research and policy away from blaming individuals and towards recognising system-level weaknesses as the primary sources of risk. According to the SEIPS model (Holden *et al.*, 2013), safe care depends on the interaction between people, tasks, tools, organisation and environment. Placing responsibility solely on the individual neglects how factors such as workload, interruptions and resource allocation constrain what staff can realistically achieve. WHO, the current global leader in patient safety, similarly emphasises this perspective. Their patient safety strategy and infection prevention guidelines underline that behavioural change and sustainable improvements require system-level change, supportive leadership, a supportive culture and adequate resources (World Health Organization, 2021).

## General discussion

The managers in our study described numerous strategies they use to improve working conditions for their staff to enable them to perform proper infection prevention behaviours. We have previously discussed our analysis in relation to the health belief model (Glanz *et al.*, 2015), a model relevant for understanding behaviours among staff. As managers, it is crucial to recognise that multiple factors can influence an individual’s actions, even when they are aware of what they “should” do. However, a limitation of this approach is that it primarily focuses on the individual, why we suggest that it should be understood in combination with the principles of social influence (Cialdini, 2009), which align with the contextualist perspective that underpins our study.

The analysis resulted in four interrelated themes: adjusting staffing and other organisational conditions to reduce workload and interruptions, cultivating a supportive team climate, strengthening knowledge and understanding among nursing staff and promoting personal responsibility. These four themes operate on different levels: the first three emphasise organisational and system-level strategies, whereas Theme 4 highlights an individualised view of responsibility. From a systems perspective, such as the SEIPS 2.0 model (Holden *et al.*, 2013), safe care is shaped by the interaction of system elements rather than by individual behaviour alone. The coexistence of these orientations in our findings points to a key tension in managerial perspectives between system-level adaptation and individual accountability. Furthermore, the contrast between Theme 4 and the other themes evokes different leadership styles. Transactional and transformational leadership styles are widely discussed in the literature and elements of both are apparent in our findings. Transformational leadership is characterised by attentiveness to staff needs, trust-building, motivation, creativity, collaboration and development (Arenas, 2019). These features align closely with the strategies described in Themes 1–3, where managers emphasised efforts to improve staff working conditions. Conversely, Theme 4 reflects aspects of transactional leadership, which prioritises responsibility and the achievement of organisational goals through strict adherence to guidelines and expectations. In Theme 4, managers underlined that hygiene routines are non-negotiable and must be upheld regardless of working conditions. Importantly, these orientations were not tied to specific managers or levels of leadership but rather co-existed within the same individuals. This reflects what the literature also describes: these approaches are not mutually exclusive but rather correlated and frequently co-exist in practice (Arenas, 2019). This illustrates the complexity of managerial work in infection prevention, where managers must both adapt to circumstances and maintain firm expectations regarding compliance. In complex healthcare environments, it is therefore not surprising that managers shift between orientations depending on context. Although few studies have directly examined how leadership relates to infection prevention behaviours, the gap noted in the WHO research agenda (Tartari *et al.*, 2025) – a recent conceptual analysis – has considered the broader role of leadership styles in shaping patient safety work (Wijaya, 2024). In this work, transformational leadership was associated with open communication, encouragement of incident reporting and continuous improvement – factors considered essential for strengthening safety culture. Transactional leadership, in contrast, was viewed as effective for promoting rule adherence and maintaining operational performance, but its contribution to fostering proactive safety behaviours was described as less consistent (Wijaya, 2024). To conclude, the transformational aspects that empower staff, build trust and strengthen collective responsibility emerge as the most desirable in patient safety work and should therefore be regarded as equally important goals in infection prevention efforts.

Managers in this study described a range of working conditions that hinder nursing staff’s infection prevention behaviours. These barriers are not unique to our context. A Cochrane synthesis identified many of the same challenges, including workload pressures, time constraints, competing demands, limited training opportunities and physical constraints. The review also highlighted clear communication, consistent managerial support and high-quality, mandatory training as key facilitators (Houghton *et al.*, 2020). Taken together, our findings align with this international evidence and underscore that sustainable improvements in infection prevention require both organisational support and strong managerial leadership.

### Study evaluations and reflections

A key strength of this study lies in the use of reflexive thematic analysis, which enabled a nuanced exploration of shared and divergent managerial perspectives, while also acknowledging the active role of the researcher in shaping interpretation. This analytic approach was particularly valuable given the limited amount of prior research on managerial perspectives in infection prevention. In line with the conceptualisation of reflexive thematic analysis, we did not use validation procedures such as inter-coder reliability, co-coding or member checks. These strategies are not congruent with the epistemological foundations of reflexive thematic analysis, where rigour is instead achieved through transparency, reflexivity and theoretical coherence (Braun and Clarke, 2024). To ensure quality, we engaged in continuous reflexive dialogue within the research team and made analytic decisions explicit throughout reporting. A methodological decision was not to distinguish between FLMs and SLMs in the analysis. Given the exploratory nature of the study and the need for more research on managerial influence in hygiene practices, as highlighted in the WHO agenda, a broad overview was prioritised over subgroup comparisons. This decision was reinforced by the impression from data collection and analysis that no clear differences were evident, likely due to their close collaboration in daily operations and the fact that several SLMs had prior FLM experience, reflected in their responses.

Given our contextualist perspective, it is not reasonable to discuss generalisability in its traditional sense. Instead, in line with Braun and Clarke (2022), we recognise the importance of describing the context of data collection, which we have aimed to do in both the methods and the reporting of data analysis to facilitate assessments of transferability. Although this study was conducted in surgical and orthopaedic units within a Swedish context, we believe that the findings are applicable to other 24/7 healthcare settings, both in Sweden and internationally and can provide valuable insights for similar organisations. In retrospect, we would have explored more deeply how managers’ personal values, experiences and perceptions of risk shape their leadership, as well as “soft” aspects such as workplace culture and climate. Although partly addressed through discussions on the psychosocial work environment, further attention to these factors could have provided a richer understanding of the context in which managers and staff operate.

### Clinical implications, suggestions for future research and conclusion

Our findings highlight several barriers to implementing and sustaining infection prevention behaviours, including high workload, frequent interruptions, staff turnover, workplace culture and variable knowledge levels. These barriers must be explicitly addressed when designing interventions, as strategies that overlook the realities of everyday work risk being unfeasible. FLMs and SLMs have complementary responsibilities and both levels need to be engaged in implementation. FLMs are well positioned to strengthen unit culture and support staff in daily practice, while SLMs can influence broader organisational structures. However, both operate under resource constraints, underscoring the need for higher-level decision-makers to prioritise working conditions as part of patient safety agendas.

Linking our findings to the WHO Global Patient Safety Action Plan, we argue that leadership development programmes should integrate infection prevention as a core component, emphasising not only compliance monitoring but also strategies to foster culture, teamwork and sustainable working conditions. This is important not only for staff well-being but also for patient safety and economic sustainability, as HCAIs prolong hospital stays and increase costs. Policymakers and organisational managers should therefore consider infection prevention as a key performance indicator in leadership and quality frameworks.

For research, future studies should move beyond general calls for “improved leadership” and test organisation-specific interventions tailored to local contexts, co-designed with both staff and managers. Systematic evaluation of implementation barriers is also needed to understand not only what works but also under what conditions improvements can be achieved. Improving infection prevention behaviours requires that the organisational factors highlighted in Themes 1–3 are in place, as these provide the foundation for behavioural change and the maintenance of safe care. Taken together, these implications are directly relevant for policymakers, organisational managers, educators and researchers, each of whom plays a critical role in advancing infection prevention as both a patient safety and workforce sustainability priority.
